# Nuclear protein accumulation in cellular senescence and organismal aging revealed with a novel single-cell resolution fluorescence microscopy assay

**DOI:** 10.18632/aging.100372

**Published:** 2011-10-16

**Authors:** Marco De Cecco, Jessie Jeyapalan, Xiaoai Zhao, Mimi Tamamori-Adachi, John M. Sedivy

**Affiliations:** ^1^ Department of Molecular Biology, Cell Biology and Biochemistry, Brown University, Providence, RI 02903, USA; ^2^ Department of Veterinary Science, University of Bologna, Bologna, Italy; ^3^ Department of Biochemistry, Teikyo University School of Medicine, Tokyo, 173-8605, Japan

**Keywords:** Aging, cellular senescence, quantitative protein assay, fluorescence microscopy, NanoOrange^®^ reagent

## Abstract

Replicative cellular senescence was discovered some 50 years ago. The phenotypes of senescent cells have been investigated extensively in cell culture, and found to affect essentially all aspects of cellular physiology. The relevance of cellular senescence in the context of age-associated pathologies as well as normal aging is a topic of active and ongoing interest. Considerable effort has been devoted to biomarker discovery to enable the microscopic detection of single senescent cells in tissues. One characteristic of senescent cells documented very early in cell culture studies was an increase in cell size and total protein content, but whether this occurs in vivo is not known. A limiting factor for studies of protein content and localization has been the lack of suitable fluorescence microscopy tools. We have developed an easy and flexible method, based on the merocyanine dye known as NanoOrange, to visualize and quantitatively measure total protein levels by high resolution fluorescence microscopy. NanoOrange staining can be combined with antibody-based immunofluorescence, thus providing both specific target and total protein information in the same specimen. These methods are optimally combined with automated image analysis platforms for high throughput analysis. We document here increasing protein content and density in nuclei of senescent human and mouse fibroblasts in vitro, and in liver nuclei of aged mice in vivo. Additionally, in aged liver nuclei NanoOrange revealed protein-dense foci that colocalize with centromeric heterochromatin.

## INTRODUCTION

Normal somatic cells, with the exception of germ cells and some stem cells, display a finite replicative capacity. This phenomenon was first described in cultured human fibroblasts [1], and is commonly referred to as replicative cellular senescence. Subsequently, these observations were extended to a wide variety of vertebrate species and cell types, and senescence is now believed to be a general property of replicative cells [2]. Cellular senescence has been studied predominantly in cell culture (in vitro), although evidence is accumulating that this process also occurs in intact organisms (in vivo) under a variety of pathological and normal conditions [3-5]. A well studied trigger of cellular senescence is the shortening of telomeres [6], but many other stimuli, most importantly the activation of oncogenes and a variety of genotoxic stresses, have also been documented [7]. The phenotypes of senescent cells affect most, if not all aspects of cellular physiology, including gene expression, chromatin organization, protein processing and metabolism [3, 8]. Given the extensive, complex, and often cell-type specific nature of these changes this remains an active area of investigation.

In vivo studies have been especially hampered by the complex phenotypes of senescent cells as well as their low abundance under most normal conditions. We, as well as others, have expended considerable effort in developing biomarkers to enable the microscopic detection of single senescent cells in tissues [9-11].

Early in vitro studies revealed fundamental changes in cellular morphology such as increased cellular volume, increased nuclear and nucleolar size, and numerous cytoplasmic changes such as prominent golgi, vacuolated cytoplasm, and large lysosomes (for reviews of early work see [2, 12, 13]). The increased lysosomal activity is believed to be the basis of the well known senescence-associated b-galactosidase (SA-b-Gal) assay [9, 14]. While senescent cells maintain a stable diploid DNA content, commensurate with their increased size both the total RNA and total protein content are increased [15-20]. The latter occurs in spite of a decrease in the overall rates of synthesis, and is believed to be caused by a decrease in turnover [21, 22] as well as unbalanced growth (ongoing macromolecular synthesis in the absence of cell division), leading to a documented adjustment of the cellular proteome [23, 24].

The accumulation of oxidized, misfolded and/or aggregated proteins with age is well documented [25, 26], but few studies have been performed at single cell resolution in the complex mosaics of functional and dysfunctional cells that comprise aged tissues. An important limiting factor has been the lack of a well developed and accepted staining method for total protein suitable for fluorescence microscopy. This is in contrast to DNA, where dyes based either on intercalation (acridine) or minor groove binding (DAPI, Hoechst) have been widely used for some time, and have enabled many high resolution as well as quantitative studies in numerous applications. While a number of sensitive staining methods have been developed for protein quantification in solution as well as on solid supports [27], these principles have not been extended and generalized for fluorescence microscopy. We report here the development of a novel method to stain and observe total protein by high resolution fluorescence microscopy, which we apply to study protein content changes in senescing human and mouse fibroblasts in vitro, and in young and aged mouse liver tissue in vivo.

## RESULTS

### Total protein content of early passage and senescent fibroblasts in culture

We first confirmed previous reports that senescent cells contain more protein using the strain of normal human diploid fibroblasts (HDF) commonly used in our lab (LF1, see Methods). Total protein was extracted from early passage, senescent, or early passage quiescent cells, and assayed using the EZQ Protein Quantitation Kit (Invitrogen, Supplemental Methods). Additionally, we fractionated whole cells into cytoplasmic and nuclear extracts, and determined their relative total protein concentrations (Supplemental Figure 1A). The values were normalized to either cell number, DNA content, or histone H2B content (Supplement Figure 1B). Over a large number of experiments we saw consistent increases in total, cytoplasmic and nuclear protein content. These data are in good agreement with previous reports [16, 28, 29].

### A fluorescence microscopy method for total protein staining and measurement

**Figure 1:**
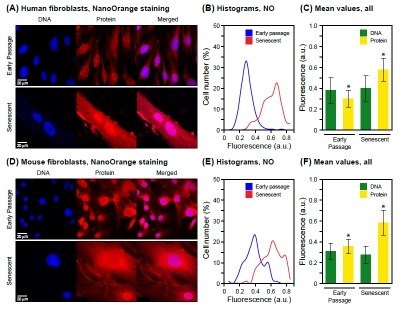
Staining of total protein in intact cells and visualization by fluorescence microscopy. **(A)** Representative images acquired with cultures of human fibroblasts (HDF). DNA was stained with DAPI (left panels) and protein was stained with NanoOrange (NO, middle panels) as indicated in Methods. Merged images are shown in the right panels. The NanoOrange signal was acquired at 560-620 nm and is shown in the red channel. Early passage cells are shown in the upper panels, and senescent cells in the lower panels. **(B)** The data for NanoOrange staining (total protein) are displayed as histograms of mean fluorescence intensity (Table 1) in arbitrary units (a.u.) on the X axis, and cell number (% of total) on the Y axis. Fluorescent images were quantified using CellProfiler software as indicated in Methods. A minimum of 1000 cells were scored for each condition (early passage, senescent). **(C)** Mean values of staining intensity in arbitrary florescence units (a.u.) and their associated standard deviations are shown for both DNA and total protein in early passage as well as senescent cells. The increase in protein signal is statistically significant (* p < 0.001). The signal for DNA does not change between early passage and senescence (p = 0.19). A minimum of 1000 cells were scored for each condition (early passage, senescent) and channel (DNA, blue; protein, red). **(D)** Representative images acquired with cultures of adult mouse tail fibroblasts (MTF). The panels are arranged and labeled as indicated in (A). **(E)** Histograms of NanoOrange staining for early passage and senescent MTF cells. The data are displayed as indicated in (B). **(F)** Mean values of DNA and total protein staining for early passage and senescent MTF cells. The data are displayed as indicated in (C). As with the HDF, the DNA signal did not change significantly (p = 0.12), whereas the increase of the protein signal was significant (* p < 0.001).

We next stained cells grown on glass cover slips using the NanoOrange dye, which was originally developed for highly sensitive biochemical detection of proteins in solution by Molecular Probes [30]. We systematically explored the parameters affecting the performance of the assay (Supplemental Figure 2), and developed a protocol that provides highly consistent staining of whole cells (Methods). Notably, our staining method is stable over a wide range of paraformaldehyde fixation times, affording excellent preservation of cellular morphological features. As expected from the biochemical fractionation experiments, stained preparations of senescent HDF observed by confocal fluorescence microscopy displayed a clear increase of both cytoplasmic and nuclear signals (Figure 1A). To extend the generality of these findings, we also investigated senescent mouse fibroblasts and observed very similar increases of cytoplasmic and nuclear protein content (Figure 1D). We used cultures of mouse tail fibroblasts (MTF) prepared freshly from adult biopsy material (Methods), which in contrast to mouse embryo fibroblasts (MEF) undergo senescence even under physiological (2.5%) oxygen levels [31], and display characteristic increases of SA-b-Gal and senescence regulators such as p16^Ink4a^ (Cdkn2a [32] and our unpublished observations).

We subsequently focused on quantitative image analyses of the nucleus. Senescence-associated changes in protein content of this compartment have not been widely studied. More importantly, the development of methods for the examination of single cells in complex tissues depends on the ability to unambiguously assign the regions of interest, which in the case of the nucleus can be done accurately by DAPI staining. To enable the high throughput necessary for statistical significance, we applied the open source software CellProfiler and CellProfiler Analyst, which allows the analysis of thousands of images through automated pipelines (Methods).

We documented a highly significant senescence-associated increase in total nuclear protein in both HDF (Figure 1B, C) and MTF (Figure 1E, F), while as expected, DNA content did not change significantly. Interestingly, the mean protein intensities increased 1.93-fold for HDF and 1.64-fold for MTF (Table 1), suggesting that the nuclei had become more dense in protein. Since the DNA signals did not change significantly, the ratio of protein to DNA intensity also increased, 1.84-fold in HDF and 1.82-fold in MTF, further confirming the increase in protein density. Hence, the senescence-associated change in nuclear protein content can be conveniently observed and documented as an increase of DAPI-normalized mean NanoOrange intensity.

**Table 1:** Summary of fluorescence microscopy determined protein data in HDF and MTF

	Mean^1^	Mean^2^	Ratio^3^	Fold-change^4^	Fold-change^5^
	Protein	DNA	Protein/DNA	Protein	Protein/DNA
Early Passage HDF	0.30	0.38	0.79	1	1
Senescent HDF	0.58	0.40	1.45	1.93	1.84
					
Early Passage MTF	0.36	0.31	1.16	1	1
Senescent MTF	0.59	0.28	2.11	1.64	1.82
^1^ The mean protein content (mean protein signal per nucleus averaged for all nuclei observed).				
^2^ The mean DNA content, calculated as for column 1 using intensity values recorded in the DAPI channel.				
^3^ The mean protein content normalized to the mean DNA content (column 1/column 2).				
^4^ Comparison of the mean protein content (column 1) between early passage and senescent cells. The value of senescent cells was normalized to that of early passage cells for both HDF and MTF.				
^5^ Comparison of the DNA-normalized mean protein content (column 3) between early passage and senescent cells. The value of senescent cells was normalized to that of early passage cells for both HDF and MTF.				

### Multiparameter immunofluorescence and NanoOrange protein staining

The utility of the NanoOrange staining of total protein would be greatly enhanced if it could be combined with immunological detection of specific proteins. Preliminary experiments were performed to test whether the NanoOrange dye would interfere with antibody detection, or whether the extensive washing of immunofluorescence protocols would decrease the dye signal. Repeated trials where the same specimens were processed in triplicates (NanoOrange alone, immunofluorescence alone, NanoOrange and immunofluorescence combined) and analyzed for all parameters with CellProfiler did not reveal any significant interference (data not shown).

**Figure 2:**
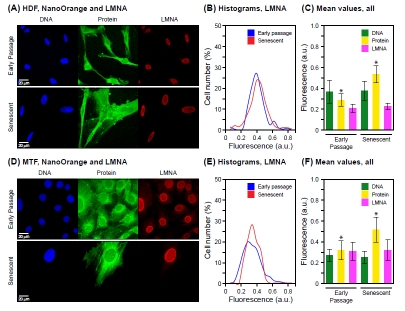
Combination of NanoOrange total protein staining with immunofluorescence microscopy Cell we stained with NanoOrange (NO) and subsequently processed for immunofluorescent detection of lamin A/C (LMNA) as indicated in Methods. (A) Representative images acquired with cultures of HDF. DNA (DAPI), total protein (NanoOrange) and LMNA (immunofluorescence) are shown in the left, middle and right panels, respectively. Note that in this figure the NanoOrange signal (acquired at 560-620 nm) is shown pseudo-colored in green, and the LMNA signal (acquired at 665 nm and above using an Alexa 647 secondary antibody) is shown in the red channel. Early passage cells are shown in the upper panels, and senescent cells in the lower panels. (B) Histograms of LMNA staining for early passage and senescent HDF cells. The data are displayed as indicated in Figure 1B. (C) Mean values of staining intensity for DNA, total protein and LMNA in early passage and senescent HDF cells. The data are displayed as indicated in Figure 1C. The increase in protein signal is statistically significant (* p < 0.001). The signal for either DNA (p = 0.16) or LMNA (p = 0.22) does not change significantly between early passage and senescence. (D) Representative images acquired with cultures of MTF. The panels are arranged and labeled as indicated in (A) above. (E) Histograms of LMNA staining for early passage and senescent MTF cells. The data are displayed as indicated in (B) above. (F) Mean values of staining intensity for DNA, total protein and LMNA in early passage and senescent MTF cells. The data are displayed as indicated in (C) above. As with the HDF, the DNA or LMNA signals did not change significantly (p = 0.14 and p = 0.13, respectively), whereas the increase of the protein signal was significant (* p < 0.001).

We subsequently investigated using these multiparameter assays major structural nuclear proteins (lamin A/C, LMNA), histones (histone H3), and proteins that have been documented to increase in senescent cells (the heterochromatin component macro H2A, mH2A). LMNA levels did not differ between early passage and senescent cells, either HDF (Fig. 2A-C) or MTF (Fig. 2D-F). In the same specimens, total protein was increased as observed previously. Thus, the increase in total protein is not a consequence of a general increase of all nuclear proteins, and conversely, the major structural nuclear component LMNA cannot account for the increase of total nuclear protein.

**Figure 3:**
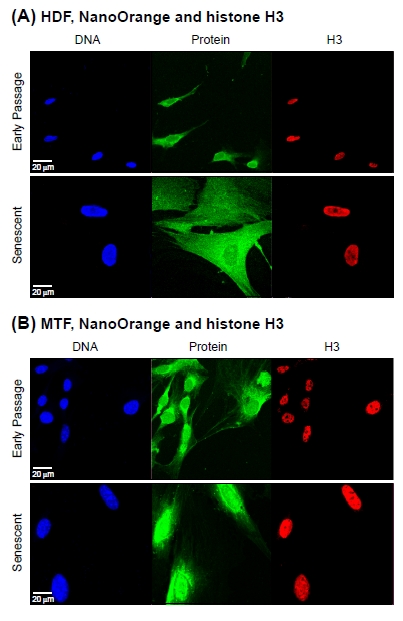
Co-staining of total protein and histone H3 Cell we stained with NanoOrange (NO) and subsequently processed for immunofluorescent detection of histone H3 (H3) as indicated in Methods. **(A)** Representative images acquired with cultures of HDF. The panels are arranged and labeled as indicated in Figure 2A. **(B)** Representative images acquired with cultures of MTF. The panels are arranged and labeled as indicated in (A) above.

The same pattern was observed in a mutiparameter assay scoring histone H3 (H3) by immunofluorescence and total protein by NanoOrange staining: H3 did not increase, while total protein did (Figure 3A, B). The lack of H3 increase would be expected from the constant DNA signal, and indicates that the increase in total protein is unlikely to be accounted for by major changes in the DNA/histone ratio of chromatin. In support, the levels of histone H2B were not observed to increase in senescent cells by immunoblotting (Supplemental Figure 1B). It should be noted that the absence of increase in LMNA or H3 was not due to effects of NanoOrange staining, since (as noted above) single parameter assays for either LMNA or H3 gave the same results.

**Figure 4:**
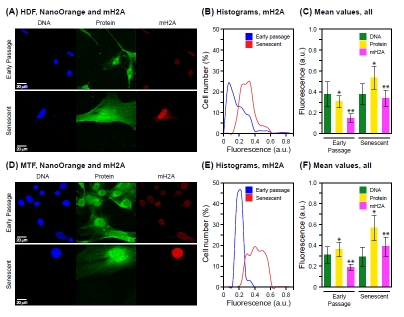
Co-staining of total protein and histone macro H2A Cell we stained with NanoOrange (NO) and subsequently processed for immunofluorescent detection of histone macro H2A (mH2A) as indicated in Methods. (A) Representative images acquired with cultures of HDF. The panels are arranged and labeled as indicated in Figure 2A. (B) Histograms of mH2A staining for early passage and senescent HDF cells. The data are displayed as indicated in Figure 2B. (C) Mean values of staining intensity for DNA, total protein and mH2A in early passage and senescent HDF cells. The data are displayed as indicated in Figure 2C. The increases of both protein and mH2A signals are statistically significant (* p < 0.001, ** p < 0.001). The signal for DNA did not change significantly between early passage and senescence (p = 0.34). (D) Representative images acquired with cultures of MTF. The panels are arranged and labeled as indicated in (A) above. (E) Histograms of mH2A staining for early passage and senescent MTF cells. The data are displayed as indicated in (B) above. (F) Mean values of staining intensity for DNA, total protein and mH2A in early passage and senescent MTF cells. The data are displayed as indicated in (C) above. As with the HDF, the increases in both protein and mH2A signals were statistically significant (* p < 0.001, ** p < 0.001), whereas the DNA signal did not change significantly (* p = 0.29).

In contrast, a multiparameter assay scoring mH2A by immunofluorescence and total protein by NanoOrange staining showed the typical increase in total protein (Figure 4A-C), as well as the previously documented [11] increase in mH2A (Figure 4D-F) in the same specimens. This further underscores the multiplexing capability of NanoOrange staining with immunofluorescence. Since mH2A is a relatively minor component of chromatin, being typically found at levels some 2-orders of magnitude below canonical histones [33], its increase is unlikely to account for a significant fraction of the total nuclear protein increase.

### Application of NanoOrange staining to tissue specimens

**Figure 5:**
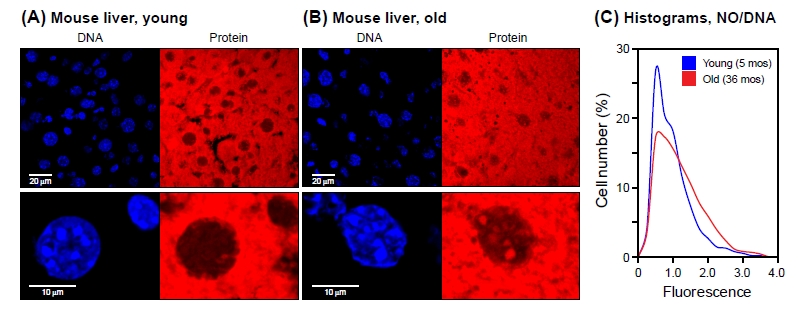
NanoOrange staining of mouse liver tissue Cryosections of liver tissue were processed and stained with NanoOrange (NO) as indicated in Methods. (A) Young (5 months old). (B) Old (36 months old). Left panels, DAPI staining; right panels, NanoOrange staining. Lower panels shown selected nuclei at high magnification (note scale bars at the left of the DAPI images). Note bright NO-staining foci in old nuclei that colocalize with DAPI-staining foci. All mice were C57Bl/6 males and were healthy at the time of sacrifice. (C) Histograms of NanoOrange staining for young and old liver tissue. Fluorescent images were quantified using CellProfiler software as indicated in Methods. The data are displayed as indicated in Figure 1B, with the exception that for each cell the protein mean intensity signal was normalized to its DNA mean intensity signal (NO/DAPI). Samples from 4 young and 4 old mice were examined, with approximately 1000 nuclei scored for each mouse. The data were pooled, resulting in >4000 nuclei being scored for each condition (young/old). The two distributions were found to be significantly different from each other (Kolmogorov-Smirnov test, p < 0.001), with a tendency for the old liver nuclei to have larger values (Wilcoxon rank-sum test, p < 0.001).

The eventual goal of the work presented here was to develop a novel single-cell bioassay to monitor age associated changes in mammalian tissues. We therefore examined liver sections obtained from healthy young (5 months) and old (36 months) male mice (Figure 5A-C). Liver tissue contains very abundant cytoplasmic proteins resulting in a bright NanoOrange signal. We focused our analysis on the nuclei, which were defined on the basis of DAPI staining. Compared to cells grown on cover slips, tissues such as liver contain nuclei oriented in 3 dimensions. Since the CellProfiler software currently cannot process z-axis stacks of images, we took a single optical section through the center of the tissue slice, and set software filters to retain only the largest objects in the DAPI channel, thus focusing the analysis on nuclei that were bisected close to their centers. This eliminates nuclei that were bisected close to one edge, and minimizes out of focus NanoOrange signals from over (or under) lying cytoplasm. All data were internally normalized to DNA content, and are expressed as ratios of mean NanoOrange to mean DAPI intensities acquired from the same optical section.

The mean protein intensity of the old nuclei was some 25% greater than that of the young nuclei (0.36 and 0.29, respectively). Notably, the two histogram distributions had different shapes: the old distribution contained a significant fraction of high intensity nuclei and commensurately fewer low intensity nuclei (Figure 5C). Given the large number of nuclei (>4000 for each condition) scored by the CellProfiler pipeline, the two histogram distributions are statistically highly significantly different. In addition, since the software keeps track of each individual nucleus, the high intensity nuclei can be extracted from the dataset and scored for additional parameters.

We also noted that at high magnification young liver nuclei displayed relatively uniform NanoOrange staining, while almost all the old nuclei contained prominent bright foci (Figure 5A,B, lower panels). These foci largely colocalized with bright foci in the DAPI channel, which in the mouse are known to demarcate regions of centromeric and pericentromeric heterochromatin. It is interesting that while the DAPI foci did not change appreciably between young and old specimens, only in the old tissues NanoOrange staining increased in these regions. This suggests that the protein density of centromeric heterochromatin increases with age.

## DISCUSSION

Morphological changes were among the first reported phenotypes of senescent cells [1]. Changes in cell shape, volume and macromolecular content (DNA, RNA, protein) were actively studied in the 1970's but have received little attention since. Unfortunately, some of the original literature is not readily accessible, such as the increase in total nuclear protein of senescent HDF, which is noted in the CRC Handbook of Cell Biology of Aging [34], but the original reference [29] is not listed on PubMed. Although the increased protein content of senescent cells in vitro is in general well accepted, whether this occurs in vivo is not known. Our objective was to investigate changes in total protein as a potential new biomarker of aging, but this necessitated first the development of a fluorescence microscopy method to detect and quantify any changes.

While the Molecular Probes Handbook of Fluorescent Probes and Labeling Technologies lists numerous reagents and procedures for the staining of proteins in solution, in gels, on blots, and even on peptide microarrays, not a single application is listed for microscopy. We were attracted to fluorescent dyes because of their greater sensitivity and dynamic range relative to absorbance-based techniques. Two general principles of fluorescence-based protein detection have been developed: reactive dyes that couple with protein amines to form fluorescent, covalent adducts, and dyes that interact non-covalently with hydrophobic regions of proteins or at a protein-detergent interface.

Dyes that couple covalently with primary amines have been widely used, including the labeling of intact or permeabilized cells in flow cytometry [35, 36], but their compatibility with paraformaldehyde fixation is problematic. This is because paraformaldehyde also reacts with amines, and thus removes the reactive groups needed for subsequent dye coupling. We tested three commonly used amine-reactive dyes (fluorescamine [37], 3-(4-carboxybenzoyl) quinoline-2-carboxaldehyde (CBQCA) [38], and ophthaldialdehyde (OPA) [39]) in conjunction with a variety of organic solvent-based coagulative fixatives (such as methanol, acetone, etc.), but were unable to develop a satisfactory method that provided both good preservation of morphology and consistent, strong staining. An additional issue with amine-reactive dyes is that they show wide protein to protein differences in staining.

Dyes that interact non-covalently with hydrophobic regions of proteins gave much more consistent results, and we eventually settled on a merocyanine dye, known by the trade name of NanoOrange (Molecular Probes, Invitrogen), that produces a large increase in fluorescence quantum yield upon interaction with detergent-coated proteins [30]. It stood out among others for its high sensitivity, wide dynamic range, insensitivity to nucleic acids, relatively equivalent staining of most proteins, and above all, ease of use and high reproducibility. It is compatible with commonly used paraformaldehyde fixation, allowing the maintenance of morphological features for high resolution imaging studies. Furthermore, it can be combined with antibody-based immunofluorescent detection of specific proteins for multiparameter assays, thus providing both specific target and total protein information in the same specimen. In this communication we present optimized protocols for these new staining methods.

Using NanoOrange staining we confirmed, using single-cell image analysis, the increase of protein in senescent HDF, and extended these observations to MTF, providing additional generality to these findings. We substantiated the increase of protein in the nuclear compartment, which has not been generally noted or investigated. We were initially interested in the nucleus for practical reasons, since it can be easily and precisely identified by DAPI staining, which also provides a reliable way to normalize the protein signals. These are important quality control considerations when working with tissues. Interestingly, our data indicate, by the increase of the ratio of mean protein intensity to mean DNA intensity, that senescent cell nuclei are more dense in protein.

Multiparameter studies combining NanoOrange staining with immunofluorescence clearly showed that the increase in nuclear protein content is not caused by a general increase in the abundance of all nuclear proteins. Neither LMNA, a major structural protein, or histones H3 and H2B, were increased. As previously documented [11] the heterochromatin component histone mH2A increased in senescent cells, but because of its relatively low abundance is unlikely to account for a significant fraction of the increase in total protein. The nuclear protein (or proteins) responsible for this phenomenon thus remain to be identified.

In mouse liver we observed, in addition to the increase in signal, a localization of NanoOrange staining into prominent foci. These foci colocalized to a large extent with DAPI foci, which are known to correspond to centromeric heterochromatin. It thus appears that in mouse liver centromeric heterochromatin becomes more protein dense with age. The extent to which this accounts for the overall increase in signal is currently under investigation. It is interesting to note that while NanoOrange foci are also often seen in HDF and MTF, they are not exclusive to senescent cells, and are unlikely to be Senescence Associated Heterochromatin Foci (SAHF) since they do not colocalize well with either DAPI or mH2A foci. It is thus possible that the increase in total protein in vitro and in vivo may proceed by different mechanisms.

The combination of NanoOrange staining with automated image analysis platforms is highly desirable. First, the inherent biological variability of the specimens makes it essential to generate a large number of observations (ideally, thousands) to produce data that are highly statistically significant. The CellProfiler software [40] allowed us to achieve this in a reasonable time frame. Second, CellProfiler can be trained to recognize a variety of subcellular structures, for example, we are developing a pipeline to score liver cells for the age-associated NanoOrange nuclear foci. Third, CellProfiler can score a large number of parameters such as granularity, texture, etc., that may not have immediate biological interpretations, but should provide useful starting points for the investigation of age-associated changes in different tissues. In this context it should be noted that the cytoplasm, which we have not explored in this preliminary report for practical reasons, is very “feature rich”, and it is likely that novel information can be mined in this area. Finally, the CellProfiler Analyst platform [41] enables interactive, machine learning-based data explorations to define and score complex and novel visual phenotypes, and even use these to distinguish small subsets of cells with specific characteristics. We believe these methods will be of wide interest to biologists in many fields in addition to aging.

## METHODS

### Cell lines, culture conditions, and fixation of specimens

LF1 is a normal human diploid fibroblast (HDF) cell strain derived from first trimester female embryonic lung tissue [42]. HDF were cultured as described [11] in an atmosphere of 2.5% O_2_, 5% CO_2_, and 92.5% N_2_. Primary adult mouse tail fibroblasts (MTF) were prepared from tail biopsies of 8-12 week old C57 mice as described [31]. MTF were cultured in Dulbecco's modified Eagle's medium (DMEM) supplemented with 10% FBS under the same atmospheric conditions as HDF. Both HDF and MTF were routinely subcultured at 1:4 dilution upon reaching 80% confluence. Under our conditions HDF reach senescence at passage 50-52, and MTF at passage 11-13. Early passage HDF used in this study were at passage 15 and MTF at passage 3. HDF were cultured until proliferation ceased (monitored by daily microscopic observation) and incubated for 2 more weeks prior to harvest. MTF were monitored at each passage using the SA-b-Gal assay [9], and harvested at passage 13 when >80% of the cells were positive. For fluorescence microscopy cells were seeded onto 22 x 22 mm glass cover slips and culture was continued for at least 24 hr. Cover slips were always harvested when cells were sub-confluent, fixed with 4% paraformaldehyde in PBS for 20 min at room temperature, and permeabilized with 0.2% Triton X-100 as described [11].

### Antibodies

The rabbit polyclonal antibody to mH2A was directly labeled with Alexa Fluor 647 using a monoclonal antibody labeling kit (Invitrogen, A-20186) as described [11]. The mouse monoclonal antibody to human LMNA was from Millipore (MAB3211), and the goat polyclonal antibody to mouse LMNA was from Santa Cruz (sc-6215). Both antibodies target a region in common between the lamin A and C proteins (amino acids 464-572). Mouse monoclonal antibody to histone H3 was from Active Motif (mAb 39763). When required, appropriate Alexa Fluor 647 conjugated secondary antibodies were obtained from Invitrogen.

### Staining of cells with NanoOrange

Staining was performed in 6-well cell culture clusters, following the procedure of Jones *et al.* [30], which was developed for the staining of proteins in solution, with some modifications. During the development of our protocol we varied several parameters (temperature and time of staining, dye concentration, effect of chaotropic agents) to investigate the robustness of the assay (see Supplemental Figure 2). We also varied the time of paraformaldehyde fixation from 5 min to 60 min and observed that this did not have a significant effect on the extent of staining (data not shown). All steps were performed at room temperature unless otherwise stated, and the specimens were protected from direct light at all times. One cover slip was placed in each well, fixed as above, and washed once with PBS containing 0.2% Triton X-100 for 2-5 min. The NanoOrange Protein Quantitation kit was purchased from Invitrogen (Cat. No. N-6666). NanoOrange protein quantitation reagent was diluted to 6.25 μl/ml with 1 x NanoOrange protein quantitation diluent and 3 ml was added to each well. The plate was wrapped in Saran Wrap and then in aluminum foil (to prevent desiccation and protect from light), and incubated at 95°C for 20 min in a hybridization oven. The plate was then allowed to cool and equilibrate to room temperature for 40 min, the staining solution was removed, the cover slips were washed 3 times for 5 min in PBS, nuclei were counterstained with 2 mg/ml DAPI in PBS for 20 min, and mounted for microscopy as described [11].

### Combination of NanoOrange staining with immunofluorescence

The NanoOrange staining protocol (above) was performed up to the DAPI counterstaining step. Following the 3 PBS washes, immunofluorescence staining was initiated at the nonspecific binding blocking step, and continued essentially as described [11]. Three different blocking buffers were used, depending on the primary antibody: human LMNA, 3% donkey serum in PBS; mouse LMNA, 10% rabbit serum in PBS; mH2A and histone H3, 4% bovine serum albumin, 2% donkey serum, 2% rabbit serum in PBS. Primary antibodies were used at saturating concentrations, and incubated for 2 hr at room temperature (human LMNA, mouse LMNA) or overnight at 4 °C (mH2A, histone H3). As with the NanoOrange staining, all steps were performed at room temperature unless otherwise stated, and the specimens were protected from direct light at all times.

### Processing of mouse liver tissue

Dissection, embedding and cryosectioning were performed as described [11]. Following the fixation and permeabilization step, the specimens were stained with NanoOrange as indicated above.

### Quantitative imaging

Images were acquired using a Zeiss LSM 710 Confocal Laser Scanning Microscope. All microscope settings were set to collect images below saturation and were kept constant for all images taken in one experiment, as previously described [11]. All images were collected at 16-bit resolution in order to maximize the dynamic range of the detected intensities. Images were analyzed with the open source software CellProfiler [40] and CellProfiler Analyst [41]. ImageJ from the NIH (http://rsbweb.nih.gov/ij/) was used to convert the ZEN format files generated by the Zeiss software into TIFF files recognized by the CellProfiler software. The analysis was automated by the development of Cell Profiles pipelines (available on request), allowing the processing of large numbers of images and recording all values in databases. For each database values from at least 500 cells were compiled. Nuclei were defined using the DAPI channel (435-485 nm emission), and the total (sum of all pixels) and mean (total/number of pixels) intensities within this region were recorded for the other fluorophores: NanoOrange (560-620 nm emission), Alexa 647 (665 nm long pass emission). NanoOrange has an excitation maximum in the range of 470-490 nm, and can thus be efficiently excited by the 488 nm laser. However, both the excitation and emission peaks are rather broad (400-550 nm and 500-650 nm), making spectral overlap a significant problem in combination with dyes in the FITC/Cy2/Alexa 488 or Rhodamine/Cy3/Alexa 555 channels. However, NanoOrange can be very effectively used in combination with red-shifted dyes such as Cy5/Alexa 647. Fluorescence values were expressed as mean values in arbitrary fluorescence units (a.u.). In experiments with mouse liver tissue the mean intensities of NanoOrange were normalized to mean DAPI intensities for each nucleus. Data were displayed as histograms with nuclei distributed into bins based on their intensity levels, and plotted with the value of each bin shown as % of total nuclei. Statistical significance (p values) were assessed using the two-tailed Student's test (t-test), unless otherwise indicated. Error bars, where shown, designate standard deviations.

## SUPPLEMENTAL DATA

Supplemental Figure 1 Protein content of whole cells and cytoplasmic and nuclear fractions assessed by biochemical in-solution assay (**A**) Cell fractionation and protein quantification were performed as described in Supplemental Materials. All values shown have been normalized to cell number and are expressed as picograms of total protein per cell. This experiment was repeated twice and data are shown as means with their respective standard deviations. Numerous other experiments were performed where protein content was normalized instead to either total DNA content or to histone H2B, whose relative levels were quantified by immunoblotting. The DNA content per cell was additionally corrected for cell cycle distribution of each culture which was determined by flow cytometry. All these experiments documented a consistent increase in total, cytoplasmic and nuclear protein content of senescent cells. The values, compiled from all experimental approaches, were in the range of 4-6-fold increase in total protein, 5-7-fold increase in cytoplasmic protein, and 3-5-fold increase in nuclear protein. In all experiments the protein content of quiescent cells did not increase, and in some cases a slight decrease was seen (as also noted by others). (**B**) The quality of the cell fractionation was assessed by immunoblotting for representative cytoplasmic (GAPDH) and nuclear (histone H2B) proteins. All lanes were leaded for equivalent cell number. WC, whole cell extract; N, nuclear extract; C, cytoplasmic extract. Note the absence, in all cases, of GAPDH signal in nuclear extracts, and a corresponding absence of H2B in cytoplasmic extracts. GAPDH signal was increased in senescent cells, whereas H2B was not. No changes were seen between early passage growing and quiescent cells. mH2A, which we have previously shown to be increased in senescent cells [11] was also included for comparison. In the experiments shown in this figure, cultures designated as Early Passage were at passage 20, and those designated as Senescent were 1-2 passages prior to complete cessation of proliferation. Quiescent cultures were prepared by serum deprivation (incubation of passage 20 cells in medium containing 0.25% serum for 48 hr prior to harvest). Quiescence (>90% 2N DNA content) was confirmed by flow cytometry.

Supplemental Figure 2 Investigation of the parameters affecting the NanoOrange staining of whole cells During the development of the optimized protocol described in Methods, we varied the relevant parameters to investigate their effect on the extent of staining and the robustness of the assay. (A) Effect of temperature. The manufacturer's protocol recommends incubation at 95°C for the optimal reaction of the dye with the protein. Incubation at 20°C did not eliminate staining but decreased it significantly (* p < 0.01). The extent of staining was normalized to the 95°C reaction. (B) Effects of chaotropic agents. We investigated whether treatment prior to staining with a chaotropic agent, such with guanidium hydrochloride (Gu-HCl, 6M, 20°C, 20 min) may increase the exposure of the proteins to the dye. Under our assay conditions this did not result in a measurable difference. The extent of staining was normalized to an equivalent incubation containing PBS only (without Gu-HCl). (C) Effect of time of incubation at 95°C. The manufacturer's protocol suggest 20 min. In our assay varying the time between 15 and 25 min did not result in a measurable difference. The extent of staining was normalized to the reaction incubated for 20 min. (D) Effect of dye concentration. The manufacturer suggests diluting the dye solution provided in the kit to 2.5 μl/ml, which they designate as the 1 x working concentration. We varied the concentration from 0.625 μl/ml to 25 μl/ml (40-fold range) and noted that the extent of staining was remarkably stable. There was a slight increase in staining between 0.625 μl/ml and 6.25 μl/ml, the values being statistically significant between 0.625 μl/ml and 6.25 μl/ml (p = 0.03) and 1.25 μl/ml and 6.25 μl/ml (p = 0.05) but not between 2.5 μl/ml and 6.25 μl/ml (p = 0.13). Staining reached an apparent plateau at 6.25 μl/ml, which we chose as our standard protocol (this is 2.5 x the concentration suggested by the manufacturer for the staining of proteins in solution). The data shown in this panel were normalized to the reaction containing 6.25 μl/ml dye. In all panels the error bars designate the standard deviations.
