# Supplementary figures and images for: Nuclear protein accumulation in cellular senescence and organismal aging revealed with a novel single-cell resolution fluorescence microscopy assay
Source: Aging (Albany NY). 2011 Oct 16;3(10):955–67. doi: 10.18632/aging.100372 (PMC3229972; doi:10.18632/aging.100372)

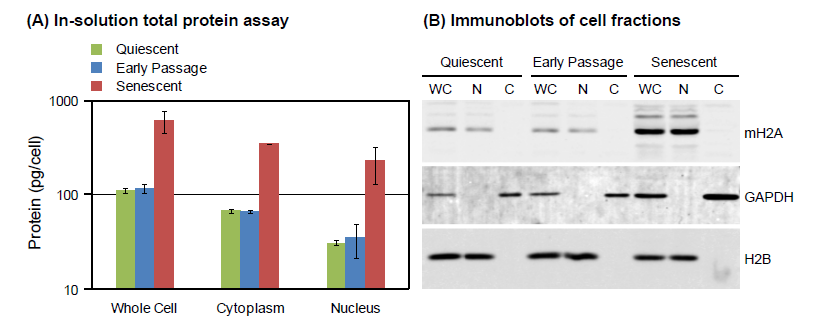

Supplement: Supplemental Figure 1 — (A) Cell fractionation and protein quantification were performed as described in Supplemental Materials. All values shown have been normalized to cell number and are expressed as picograms of total protein per cell. This experiment was repeated twice and data are shown as means with their respective standard deviations. Numerous other experiments were performed where protein content was normalized instead to either total DNA content or to histone H2B, whose relative levels were quantified by immunoblotting. The DNA content per cell was additionally corrected for cell cycle distribution of each culture which was determined by flow cytometry. All these experiments documented a consistent increase in total, cytoplasmic and nuclear protein content of senescent cells. The values, compiled from all experimental approaches, were in the range of 4-6-fold increase in total protein, 5-7-fold increase in cytoplasmic protein, and 3-5-fold increase in nuclear protein. In all experiments the protein content of quiescent cells did not increase, and in some cases a slight decrease was seen (as also noted by others). (B) The quality of the cell fractionation was assessed by immunoblotting for representative cytoplasmic (GAPDH) and nuclear (histone H2B) proteins. All lanes were leaded for equivalent cell number. WC, whole cell extract; N, nuclear extract; C, cytoplasmic extract. Note the absence, in all cases, of GAPDH signal in nuclear extracts, and a corresponding absence of H2B in cytoplasmic extracts. GAPDH signal was increased in senescent cells, whereas H2B was not. No changes were seen between early passage growing and quiescent cells. mH2A, which we have previously shown to be increased in senescent cells [11] was also included for comparison. In the experiments shown in this figure, cultures designated as Early Passage were at passage 20, and those designated as Senescent were 1-2 passages prior to complete cessation of proliferation. Quiescent cultures were prepared by seru [file aging-03-955-s001.tif]

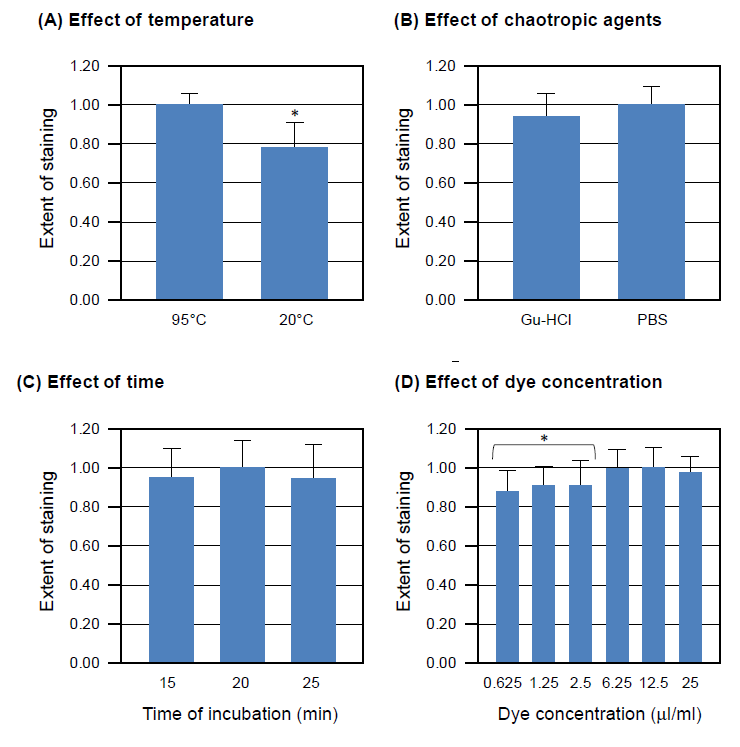

Supplement: Supplemental Figure 2 — During the development of the optimized protocol described in Methods, we varied the relevant parameters to investigate their effect on the extent of staining and the robustness of the assay. (A) Effect of temperature. The manufacturer's protocol recommends incubation at 95°C for the optimal reaction of the dye with the protein. Incubation at 20°C did not eliminate staining but decreased it significantly (* p < 0.01). The extent of staining was normalized to the 95°C reaction. (B) Effects of chaotropic agents. We investigated whether treatment prior to staining with a chaotropic agent, such with guanidium hydrochloride (Gu-HCl, 6M, 20°C, 20 min) may increase the exposure of the proteins to the dye. Under our assay conditions this did not result in a measurable difference. The extent of staining was normalized to an equivalent incubation containing PBS only (without Gu-HCl). (C) Effect of time of incubation at 95°C. The manufacturer's protocol suggest 20 min. In our assay varying the time between 15 and 25 min did not result in a measurable difference. The extent of staining was normalized to the reaction incubated for 20 min. (D) Effect of dye concentration. The manufacturer suggests diluting the dye solution provided in the kit to 2.5 μl/ml, which they designate as the 1 x working concentration. We varied the concentration from 0.625 μl/ml to 25 μl/ml (40-fold range) and noted that the extent of staining was remarkably stable. There was a slight increase in staining between 0.625 μl/ml and 6.25 μl/ml, the values being statistically significant between 0.625 μl/ml and 6.25 μl/ml (p = 0.03) and 1.25 μl/ml and 6.25 μl/ml (p = 0.05) but not between 2.5 μl/ml and 6.25 μl/ml (p = 0.13). Staining reached an apparent plateau at 6.25 μl/ml, which we chose as our standard protocol (this is 2.5 x the concentration suggested by the manufacturer for the staining of proteins in solution). The data shown in this panel were normalized to the reaction containing 6.25 μl/ml dye. In [file aging-03-955-s002.tif]
